# Variability of the Structural Coloration in Two Butterfly Species with Different Prezygotic Mating Strategies

**DOI:** 10.1371/journal.pone.0165857

**Published:** 2016-11-10

**Authors:** Gábor Piszter, Krisztián Kertész, Zsolt Bálint, László Péter Biró

**Affiliations:** 1 Institute of Technical Physics and Materials Science, Centre for Energy Research, Budapest, Hungary; 2 Hungarian Natural History Museum, Budapest, Hungary; University of Sussex, UNITED KINGDOM

## Abstract

Structural coloration variability was investigated in two Blue butterfly species that are common in Hungary. The males of *Polyommatus icarus* (Common Blue) and *Plebejus argus* (Silver-studded Blue) use their blue wing coloration for conspecific recognition. Despite living in the same type of habitat, these two species display differences in prezygotic mating strategy: the males of *P*. *icarus* are patrolling, while *P*. *argus* males have sedentary behavior. Therefore, the species-specific photonic nanoarchitecture, which is the source of the structural coloration, may have been subjected to different evolutionary effects. Despite the increasing interest in photonic nanoarchitectures of biological origin, there is a lack of studies focused on the biological variability of structural coloration that examine a statistically relevant number of individuals from the same species. To investigate possible structural color variation within the same species in populations separated by large geographical distances, climatic differences, or applied experimental conditions, one has to be able to compare these variations to the normal biological variability within a single population. The structural coloration of the four wings of 25 male individuals (100 samples for each species) was measured and compared using different light-collecting setups: perpendicular and with an integrating sphere. Significant differences were found in the near UV wavelength region that are perceptible by these polyommatine butterflies but are invisible to human observers. The differences are attributed to the differences in the photonic nanoarchitecture in the scales of these butterflies. Differences in the intensity of structural coloration were also observed and were tentatively attributed to the different prezygotic mating strategies of these insects. Despite the optical complexity of the scale covered butterfly wings, for sufficiently large sample batches, the averaged normal incidence measurements and the averaged measurements using an integrating sphere are in agreement.

## Introduction

Variation exists within all populations of living organisms [1]. Phenotypic plasticity is particularly strong in insects [2]. This is the basis of natural selection, where individuals who differ in phenotypes have different survival and reproduction chances: individuals with the appropriate trait may survive and reproduce more successfully than individuals with other, less suitable attributes; thus, the population is able to evolve. Reproductive success can also be determined by sexual selection, where one gender (most often the female) chooses a mate based on phenotypic traits [3,4]. This sexual selection is often associated with sexual dimorphism [5] and in some cases may lead to traits that are disadvantageous for the survival of the individual [6]. Sexual dimorphism is frequently found in butterflies, where long-range visual signals enhance the chances of identifying a suitable mate [7] for fast flying species. Iridescent signals, which are conspicuous and visible from a large distance during the wing movement of butterflies, may prove very efficient in achieving this goal [8]. Such colors have structural origin, in other words, they are based on photonic nanoarchitecture, a special case of nanocomposites that manipulate the propagation of light [9].

Structural colors are often found in Blue butterflies (Polyommatini, Polyommatinae, Lycaenidae, Lepidoptera). In a recent study, we showed that the structural colors of the males of nine polyommatine butterfly species living in the same type of habitat are species-specific and arise from photonic nanoarchitecture that exhibit species-specific nanostructure [10,11]. The color of the wings constitutes an important sexual communication channel; therefore, it is subjected to strong evolutionary pressure because individuals with the “wrong” color have a lesser chance to transmit their genes to offspring. The species-specific wing coloration is detected by conspecific individuals living in the same habitat; consequently, the eyes of these animals are capable of detecting the small color differences between co-habiting species. This is achieved using an additional visual pigment (compared to human eyes) that is sensitive to the violet to near UV wavelength range [12].

The structural colors of butterfly wings are increasingly the focus of attention in physics, materials science [9,13–19], and biology [8,20–23]. However, studies focusing on the biological variability of the structural coloration are lacking. Most frequently, spectral or structural characterization is conducted using only one or a few individuals. We investigated the variability of structural coloration in the case of two common polyommatine species living in Hungary in the same type of habitat. These species use their blue coloration for conspecific recognition but display differences in prezygotic strategy; the male individuals of *Polyommatus icarus* are patrolling [24], whereas *Plebejus argus* are lekking [25] in their habitats. The availability of information on the normal biologic variability of the structural coloration within a population makes possible the comparison of the color of populations that are separated by large geographical distances, often living in very different climatic conditions, or which have been purposely subjected to a certain type of experimental manipulation.

For 25 male individuals of both species, the color of all four wings was characterized spectrally using two different measurement setups, thus providing 200 measurements for each species. As butterfly wings are optically complex objects—colored by a “mosaic” of microscopic scales [9]—and spectral data are taken by human operators, two independent series of measurements for each species were performed and compared to explore the reproducibility and variability of the different measurement setups (integrating sphere and normal incidence) and to distinguish between the variation arising from the measurement conditions and from the biological variability.

The structural color of the wings is generated by a photonic crystal-type nanoarchitecture in the volume of the wing scales. This photonic nanocomposite is composed of a chitin matrix with periodically embedded air holes with a characteristic size range of a few hundred nanometers. The characteristic periodicity of the components and the refractive index contrast between them result in wavelength-selective reflection in the blue region, which is the source of the vivid structural coloration [9]. Light of other colors, which is able to penetrate the photonic nanoarchitecture, is multiple scattered and is finally absorbed by the pigments (melanin) in the structure.

However, the aspect of the wings is not only affected by the properties of the photonic nanoarchitecture, the shape and the arrangement of the scales are also significant; therefore, multiple size ranges are implicated in the generation of the reflected color [20]. Although the structural color is generated by the nanostructure in the cover scales (which can be curved by itself [26] or even fragmented [27,28]), the final visual result can be changed by the long-range order or disorder of the nanostructure in the scales. The stacking of the scales can modify the overall optical properties by multiple scattering [29]. There are cases where the structurally colored scales are covered by nanostructured but transparent scales, enhancing the scattering [13]. The wing membrane curvature due to veins also contributes to the reflection broadening. Finally, in several species, the light reflected by the scales is scattered on dense hairs covering the wings. One or more of these factors may contribute to the measured wing reflectance when the two different measurement setups are applied.

To characterize the variability of the wing coloration, the variance of the spectral parameters (see details in Materials and methods) were determined for two polyommatine species. In papers focusing primarily on structural coloration, two measurement setups (perpendicular and integrating sphere detection) are frequently used. These methods were used in this study, and the measurements were performed twice by two operators independently in the same lab using the same instrumentation. An additional reason for comparing these two methods is that the method using the integrating sphere requires the removal of the wings from the body of the butterfly, a procedure that should be avoided for precious exemplars in museums or private collections, whereas the normal incidence measurements can be conducted without harming the museum exemplars [11,30]. The results were analyzed using histograms to show the distribution of the measured parameters. A supplementary method using tridimensional color space (introduced in [11]), which is based on the color vision of these polyommatine species and shows how they can discriminate different hues of blue, is discussed in S1 File.

## Materials and Methods

### Animals and Collections

The species *Polyommatus icarus* (Rottemburg, 1775) (vernacular English name: Common Blue) is one of the most widespread Blue butterflies of the Eurasian landmass and is distributed from the Pacific to Atlantic coasts. The imagines fly in two or three annual generations in open fields and males intensively patrol for females in their habitat, which is usually mesophilous meadow in extensive or intensive human usage. The species *Plebejus argus* (Linnaeus, 1758) (vernacular English name: Silver-studded Blue) has an identical distribution and is similarly common in Hungary. *P*. *argus* inhabits the same habitat type and has two or three annual generations, but the males are sedentary, perch on grass tips, conduct sporadic flights patrolling 1–2 meters and then return to their lekking place. Consequently, individuals of *P*. *icarus* are more widespread in their habitats and can be recorded anywhere, while *P*. *argus* males aggregate in some suitable places often around or in close vicinity to the larval host plants, sometimes displaying extremely high individual numbers [31].

All of the specimens used for the measurements conducted at the Institute of Technical Physics and Materials Science were captured in the same year (2014 July) from the same field at the edge of the settlement of Érd, Hungary (47°22'21"N, 18°54'13"E). Immediately after capture, the insects were killed by freezing them in the laboratory without using any chemicals. The specimens were stored individually in closed boxes in the dark to prevent photobleaching. Prior to the optical measurements, the four wings were removed from the body. Each individual was carefully checked for taxonomic identity. In the samples used for optical measurements, both species were represented by 25 male specimens collected from the same mesophilous habitat. All samples were stored in the special insect collection of the Institute of Technical Physics and Materials Science.

### Measurement methods

To make the normal incidence measurements and the integrating sphere measurements equally possible, the four wings of each butterfly were removed to obtain 100 samples of both species. No museum collection would agree to such a massive destruction of valuable samples. Therefore, this type of detailed investigation comparing the two measurements methods was conducted for the first time. No other preparation of the samples was required.

**Fig 1:**
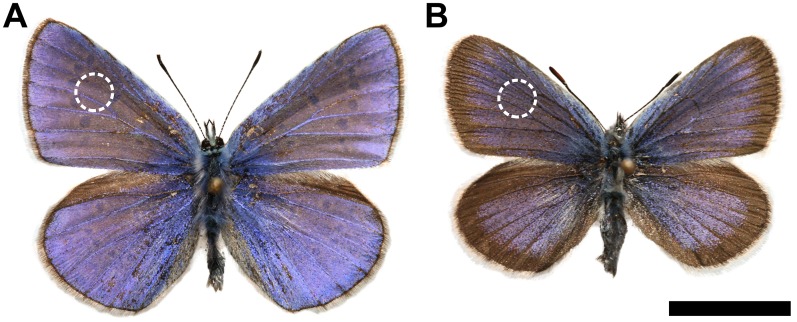
Color photographs of male Blue butterflies. (A) *Polyommatus icarus* (Common Blue) and (B) *Plebejus argus* (Silver-studded Blue) specimens are shown. The dashed circles show the location of the integrating sphere measurements. The measurement location is marked only on the left forewings. Scale bar: 10 mm.

For the characterization of optical reflectance, we used a modular, fiber optic Avantes HS-1024 TEC UV-VIS-NIR spectrophotometer (Avantes BV, Apeldoorn, The Netherlands). The Avantes diffuse tile (Avantes WS-2) was used as a white standard. To simulate the differences in measurements conducted in different laboratories, each sample was measured by two operators. The measurements by the two operators were separated in time but were performed using the same instrumentation and the same experimental protocol. Two sets of measurements were performed. In the perpendicular setup, both the illumination and detection are close to perpendicular to the sample plane (wing plane). The standard Avantes perpendicular reflection probe (Avantes FCR-7UV200-2-ME-SR) is cylindrical and is composed of 6 illumination fibers that are uniformly distributed on the circumference. Reflected light collection is performed through the central fiber; only the light reflected in the vicinity of the incident beams is collected. As the measurement time is typically shorter than a second, this is a high-yield method. The disadvantage of the method is that deviations in the incident angle from normal may generate variations in both spectral position and intensity. When using the integrating sphere (Avantes AvaSphere-50), a larger spot is illuminated than with the normal incidence measurement (see Fig 1), and the light reflected under all angles in the whole hemisphere is collected. In this way, an average reflectance is determined. Our previous investigations [27] confirm the necessity of these two methods.

After completing the measurements, a 10-point moving average filter was applied to every spectrum to eliminate high-frequency noise. The main characteristic feature on these blue wings is the reflectance peak at approximately 400 nm. On every spectrum, we determined the intensity of the peak and the wavelength at that point. We calculated the average of the peak position and peak intensity for each species and the deviation of individual samples from these values. The representation was completed in form of histograms (OriginLab OriginPro 2015).

To compare the iridescence of the wings of *P*. *icarus* and *P*. *argus*, spectrogoniometric measurements were performed using the same Avantes spectrometer in a setup we described previously [27]. This setup, in contrast to the standard Avantes reflection probe, uses single fibers for illumination and light collection (Avantes FC-UV800-2) and allows decoupling of the incidence direction of light from the direction under which the reflected light is collected. The goniometric stage allows the precise setting of the angle of the sample stage with respect to the fibers. The illuminating fiber and the collecting fiber were placed as close to each other and to the sample normal as their physical dimensions allowed. The sample plane was then tilted from -55° to 55° in steps of 5°, and a full spectrum was recorded at each position. Finally, all the spectra were plotted together in a 3D graph.

A high-resolution LEO 1540 XB scanning electron microscope (SEM) was used for the investigation of the wing scales. Samples investigated by SEM were prepared using a previously described procedure [27].

## Results

### Wing coloration and reflectance spectra

**Fig 2:**
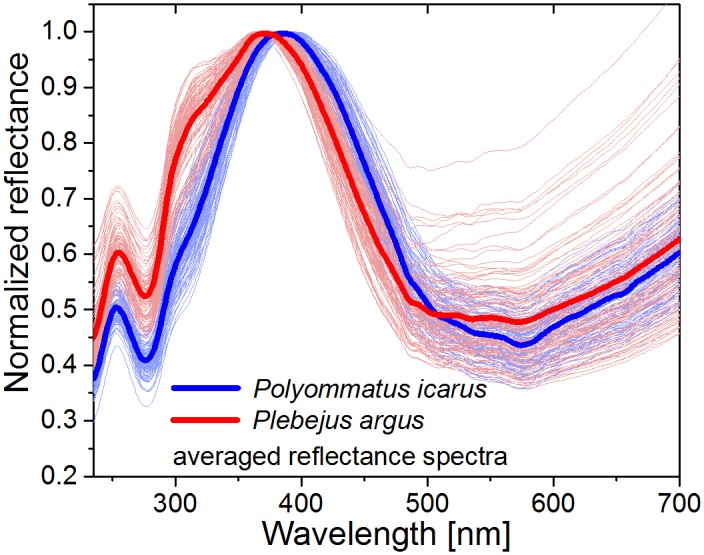
Species-specific normalized reflectance spectra (measured by integrating sphere) of *Polyommatus icarus* and *Plebejus argus*. Spectra averaged over 100 wing samples for each species are shown in bold lines, the normalized raw spectra of the wings (n = 100 each species) are plot in the background. Note the wavelength difference of the main reflectance peak and the shoulder of *P*. *argus* at approximately 320 nm.

*Polyommatus icarus* and *Plebejus argus* males have similar structural blue coloration—as observed by the naked human eye—on the dorsal surface of their wings, as shown in the photographs in Fig 1A and 1B. This blue color is generated by the so-called ‘pepper-pot’ type photonic nanoarchitecture [32], which is typical for polyommatine Blues [11]. The reflectance data on the dorsal side of all four wings of the 25 male individuals measured using the integrating sphere setup were averaged by species. The curves were normalized to the main reflectance peaks in the blue to facilitate comparison. Fig 2 shows the characteristic reflectance spectra of the two species: both species have a distinct peak at approximately 400 nm in addition to characteristic color differences. There is a small (~15 nm) difference between the wavelength of the averaged maxima in the blue, and compared to *P*. *icarus*, the averaged spectrum of *P*. *argus* males has an additional shoulder at 320 nm, showing that the wing coloration is species-specific.

**Fig 3:**
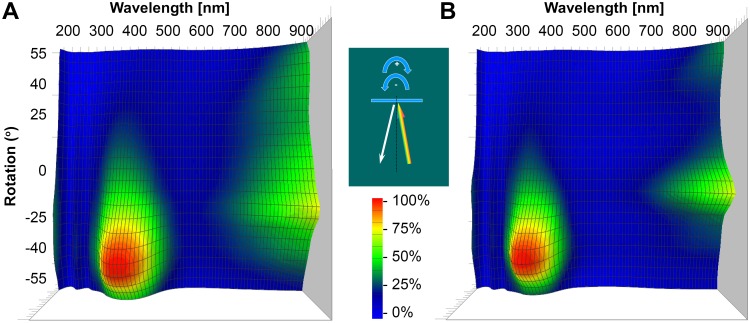
Spectrogoniometric characterization of a *P*. *icarus* (A) and of a *P*. *argus* (B) wing. Individual spectra recorded in steps of 5° are plot as a color-coded 3D surface. In the central inset, the measurement geometry is shown schematically. The illuminating beam is shown having rainbow colors, while the reflected beam is white. The iridescent behavior of the measured wings is similar.

The spectrogoniometric measurements in Fig 3 show that both *P*. *icarus* and *P*. *argus* have iridescent scales because the measured color of the wings changes with the angle. These results indicate that the measurements performed with the standard normal probe may be affected by a larger error margin if single measurements are compared than the measurements performed with an integrating sphere. However, the full spectrogoniometric characterization of several tens of samples is extremely demanding; therefore, the use of an integrating sphere may be a good compromise.

**Fig 4:**
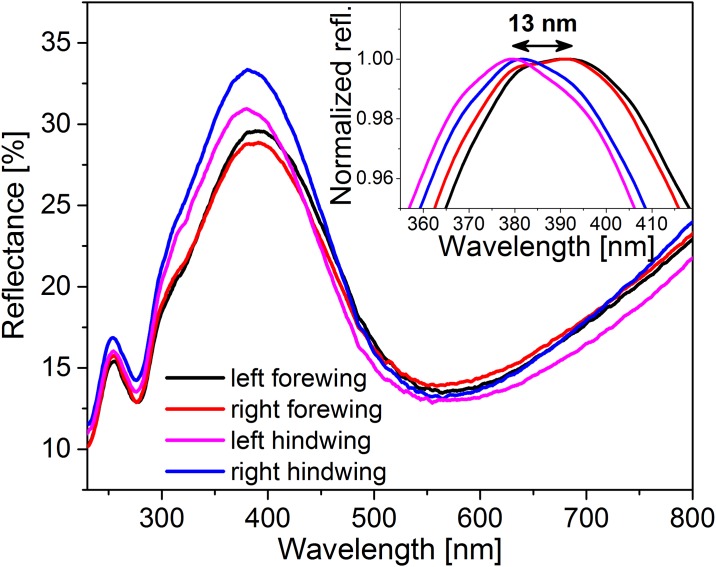
Comparison of the reflectance curves measured on the four wings of a male *P*. *icarus* butterfly using the integrating sphere. The inset in the upper right corner shows the spectral differences in the position of the normalized maxima.

Due to the way in which the macroscopic color of butterfly wings is generated from microscopic scales, the wings of the same butterfly may exhibit differences. To illustrate this characteristics, the reflectance curves of the four wings of one of the male *P*. *icarus* butterflies measured using the integrating sphere method are shown in Fig 4.

### Accuracy and reproducibility

**Fig 5:**
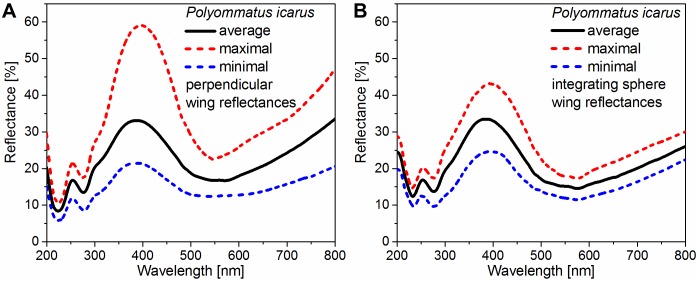
Averaged reflectance spectra of *Polyommatus icarus* measured using the two optical setups. The results of the (A) perpendicular and (B) integrating sphere light-collecting setups are shown. The red and blue curves show the recorded maximal and minimal wing reflectance (n = 100 each), respectively. The average of all measurements (drawn in black) is shown between the red and blue curves.

Fig 5 shows the averaged reflectance spectra of *Polyommatus icarus* specimens without normalization to illustrate the differences of the two light-collecting methods. Fig 5A and 5B show the measured spectra using perpendicular and integrating sphere light collection, respectively. The red and blue curves indicate the recorded maximal and minimal wing reflectance. The other spectra fall between these curves but are not shown for clarity. In the case of the perpendicular measurement setup (Fig 5A), higher variation of the peak amplitude can be observed than with integrating sphere detection (Fig 5B). The averaged amplitudes over 100 samples (black curves) are identical for both light-collecting methods, and the spectral positions (normal: 386 nm and integrated: 385 nm) differ by only 1 nm.

**Fig 6:**
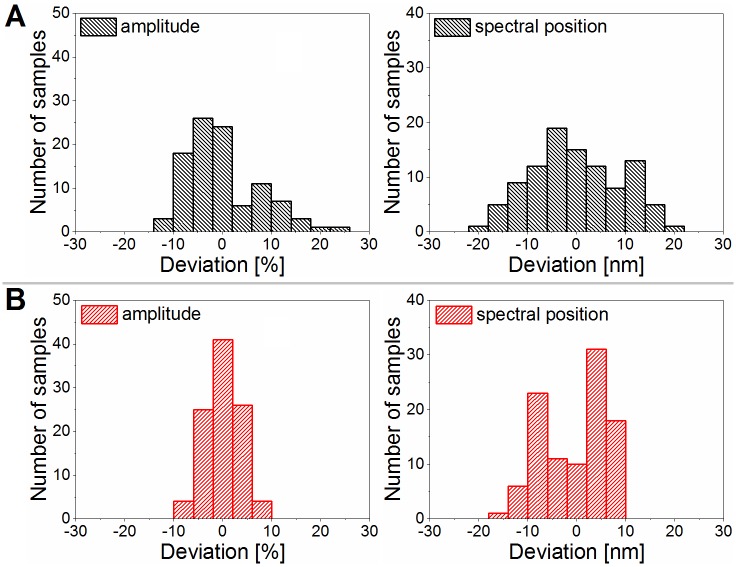
Histograms of amplitude and spectral position measured by the two optical setups. (A) The perpendicular measurement graphs exhibit a broader scatter compared to (B) the integrating sphere results, indicating that the perpendicular illumination and detection results in higher variance in the case of textured optical surfaces (n = 100 each).

Based on the averaged spectra shown in Fig 5, the averaged parameters of the amplitude and the spectral position of the peaks were determined for both measurement setups, and the deviations of the two quantities were calculated for each specimen. Using these averaged and calculated deviation parameters, histograms of the amplitude and spectral position were generated for both measurement setups (Fig 6). The histograms of both parameters are broadened when using the perpendicular measurement setup (Fig 6A) compared to the integrating sphere results (Fig 6B). The broadened amplitude histogram is in accordance with the larger deviation of the amplitudes for the perpendicular measurement in Fig 5.

**Fig 7:**
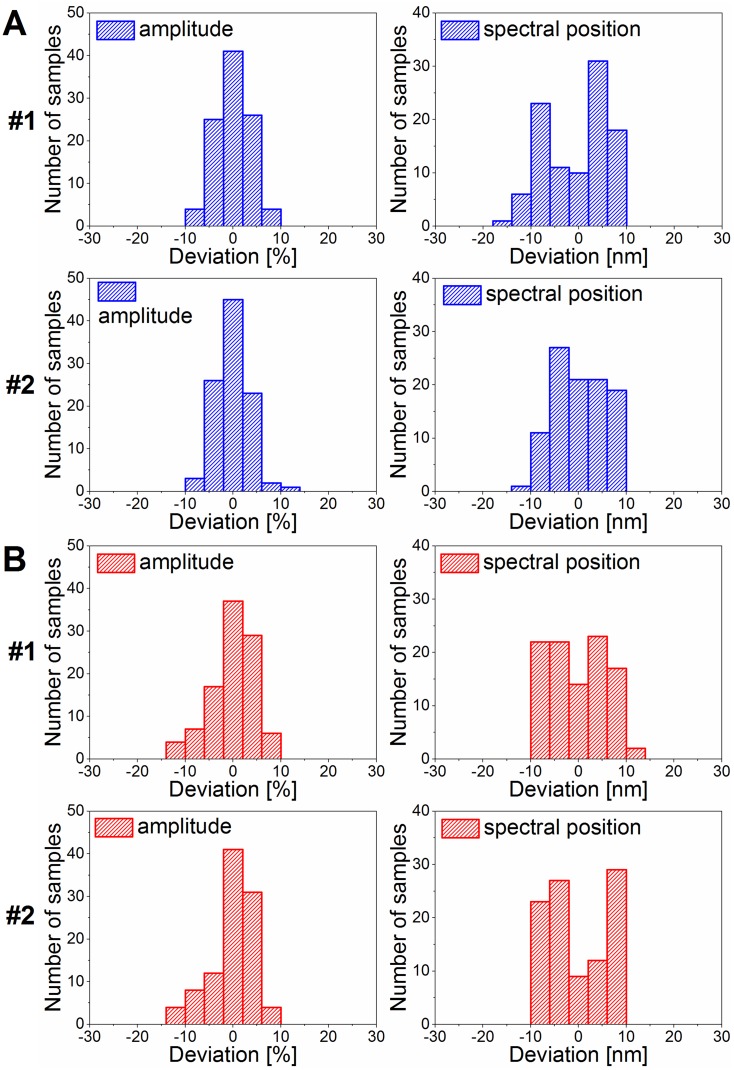
Histogram results of the spectral parameters measured using the integrating sphere by two operators. Both (A) *Polyommatus icarus* (blue) and (B) *Plebejus argus* (red) are shown. The histograms of the amplitude and spectral position measured by the different operators are similar (n = 100 each).

For complex optical surfaces, such as butterfly wings, the results of the reflectance measurements may also show differences between laboratories. To quantify these differences, two sets of measurements were conducted independently by two different operators at different times using the same equipment and the same protocol. The measured data were compared to analyze the reproducibility of the color measurements. Histograms were generated from the peak parameters of the reflectance spectra measured with the integrating sphere setup for the two users (Fig 7). The comparison of the two measurement methods using the chromaticity diagram of polyommatine butterflies (introduced in [11]) is shown in S1 Fig.

**Fig 8:**
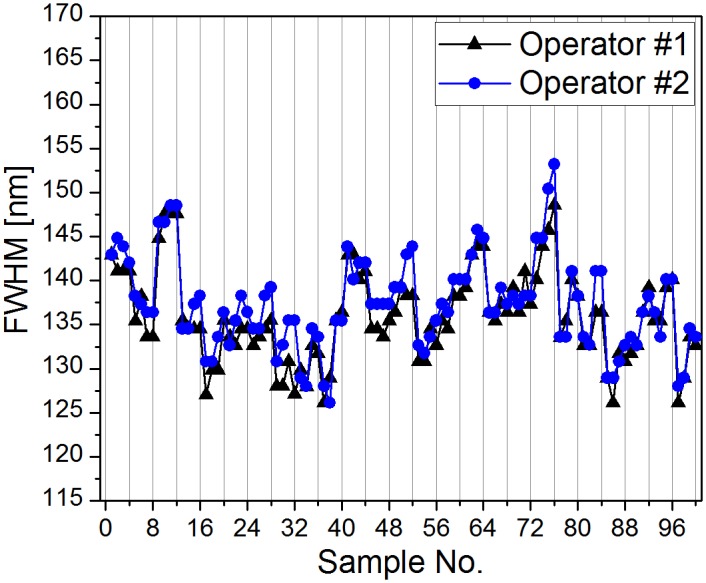
Full width at half maximum results of the main reflectance peak in the two independent measurements of *Polyommatus icarus* males. The high reproducibility of integrating sphere light collection is clearly observed (n = 100 each).

In addition to the two parameters shown above, the FWHM variance of the blue reflectance peak measured with the integrating sphere was also investigated by comparing the two sets of data. Fig 8 shows the FWHM values of the main peaks of *P*. *icarus* males, where the same set of 100 samples was measured by two independent operators. Despite the variation of the characteristics of the individual samples, the two curves corresponding to the independent measurements by the two operators are similar, which indicates good reproducibility of the individual measurements.

### Color intensity and variability

**Fig 9:**
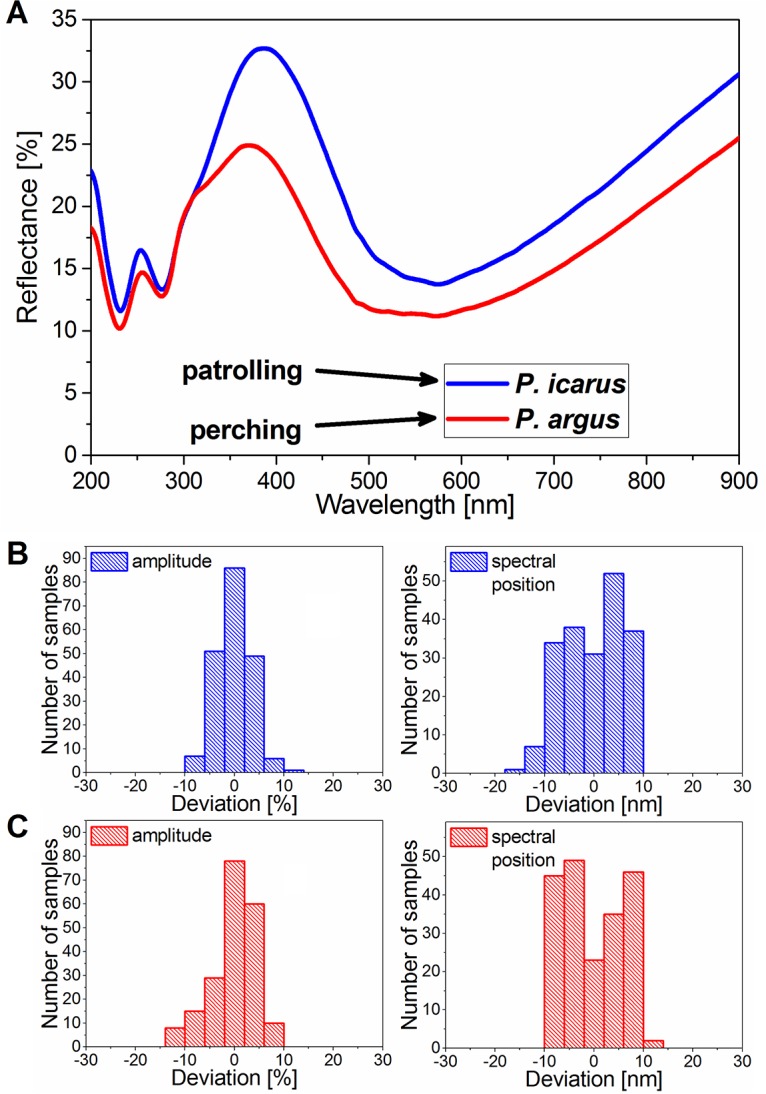
(A) Averaged reflectance spectra and histograms of the amplitude and spectral position deviations of (B) *Polyommatus icarus* and (C) *Plebejus argus* males measured with the integrating sphere light-collection setup (n = 200 each). (A) In addition to the spectral differences, such as the peak position shift and the shoulder in the *P*. *argus* spectra (shown in Fig 2), an intensity difference between the two species can be observed. (B, C) For both species, the distributions of the peak amplitude deviations have Gaussian-like shapes, while the spectral position distributions show a sharp cut-off at ±10 nm deviation, indicating that no individuals with outlier color were observed.

To investigate the intensity characteristics of *Polyommatus icarus* and *Plebejus argus*, the 100 reflectance spectra measured using the integrating sphere were averaged by species without normalization and were plotted (Fig 9A). The two averaged curves reveal the reflected intensity differences: the *P*. *icarus* males have enhanced reflectivity in the blue wavelength region (33%) compared to *P*. *argus* males (24%), resulting in a brighter blue color of the wings. This is in accordance with the naked eye observations, as shown in Fig 1.

The averaged peak position and intensity parameters were calculated from the reflectance curves measured (by both operators, n = 200) using integrating sphere light collection, and the deviations were calculated for each species. Fig 9B and 9C show the histograms of the peak amplitude and spectral position for the two species. The distributions of the peak amplitude deviations show Gaussian-like shapes, as expected. In the case of the spectral position distributions, a sharp cut-off at ±10 nm deviation is observed for both species, indicating that the probability of the occurrence of individuals with outlier color is very low.

**Fig 10:**
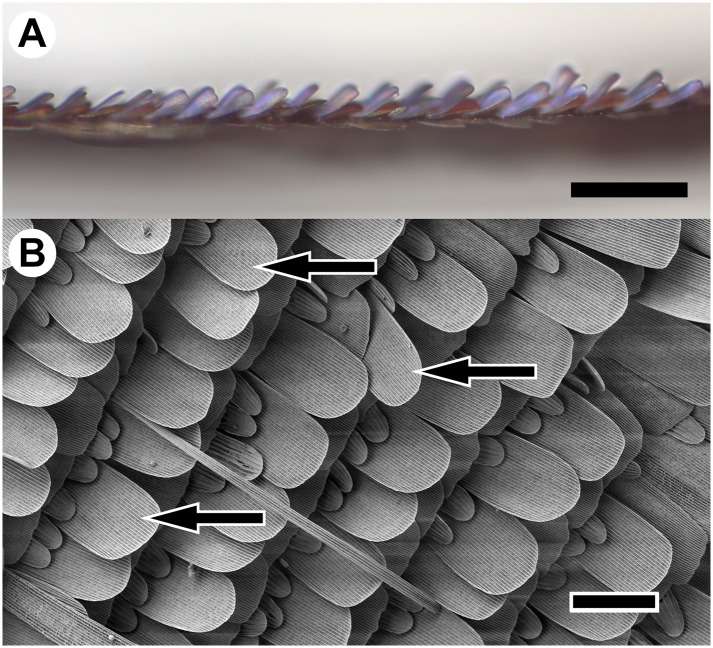
Arrangement of the scales on the dorsal wing surface. (A) Optical microscope image of a *Polyommatus icarus* wing section showing the angle of the scales to the wing membrane. Scale bar: 250 μm. (B) SEM image showing the complex arrangement of different scale types: a few degrees of rotation around the cover scale axis can be observed (some examples are indicated by arrows). Scale bar: 100 μm. Both factors contribute to the variability of the structural coloration and to the larger deviation of the perpendicular data.

Optical microscopy and SEM images of the wing scales of *P*. *icarus* are shown in Fig 10 to illustrate the arrangement of the wing scales with respect to the wing membrane (Fig 10A) and the way in which the cover scales producing the structural color are arranged in the wing plane (Fig 10B). Although the scales are mostly arranged in an orderly fashion, some of the scales (indicated by arrows) exhibit some disorder in their positions. This is equivalent to slightly different incidence conditions for the illuminating light.

## Discussion

### Measuring iridescent wings

Structural colors, in contrast to colors generated by pigments, usually exhibit a certain degree of iridescence, which requires the color measurement to account for effects that may arise from changes in the angle of incidence of the incoming light on the reflecting surface [33]. The individual scales producing the coloration of butterfly wings have typical dimensions in the range of 100 μm (Fig 10). Each of these scales is produced individually by a different cell during an only partially understood process of self-assembly [34]. Individual structural variations of few tens of nanometers cannot be excluded. These variations are sufficient to shift the color of the photonic nanoarchitecture because the size of the characteristic building elements in the case of Lycaenid butterflies is less than 100 nm [10]. As a consequence, even the wings of the same butterfly may exhibit some differences in color, even when measured with an integrating sphere, as shown in Fig 4. Therefore, when rigorous comparisons are to be made, for example, between populations of the same species living in different geographic locations or for butterflies subjected to experiments in the laboratory, which may modify their coloration, measurements on a statistically relevant number of individuals are recommended. As shown in Fig 5, despite the larger range of variation of the normal incidence measurements and the larger deviations from average (Figs 6 and 7) compared to the integrating sphere data, when the average of 100 data sets was calculated, the spectral position difference was only 1 nm and the intensity values were coincident within 1%.

The careful comparison of the normal incidence reflectance with that measured using an integrating sphere shows that the integrating sphere setup is less sensitive to accidental variation during the measurement of a complex optical object, such as a butterfly wing (Fig 10). However, it is a measurement that requires the removal of the wing from the body of the butterfly. This destructive sample preparation is prohibitive in many cases. Our measurements show that even in these cases, it is possible to obtain reliable data (by normal incidence measurements) if a sufficient number of samples are used (see Fig 5).

### Species-specific wing coloration

The dorsal cover scales of *Polyommatus icarus* and *Plebejus argus* males have blue structural coloration that appears to have a similar hue to human observers (Fig 1). We reported previously [11] that the structural coloration of Lycaenids living in the same type of habitat is generated by a complex photonic nanoarchitecture, which has some similarities for all nine Lycaenid species we investigated in addition to some species-specific features. The general structure of these pepper-pot-type nanoarchitectures [32] can be described as a stack of perforated multilayers (for a schematic drawing, see Fig 3 in [35]). The spectral position of the reflectance maximum of several Lycaenid species possessing similar photonic nanoarchitectures was found to be correlated with the perforation factor [35]. The perforation factor is understood as the ratio of the missing area and the total area in the top scale layer observed in the SEM images. On the other hand, the detailed investigation of the structural elements forming the photonic nanoarchitecture (revealed by correlated SEM and TEM images), in combination with artificial neural network software, showed that on the basis of these characteristic structural elements, it is possible to identify the butterfly species with an accuracy of 91% [11].

As one may observe in Fig 2, despite the biological variation of the spectra characterizing a certain specimen, the two families of curves for *P*. *icarus* and *P*. *argus* are well separated in the region of the reflectance maximum; consequently, the averaged reflectance spectra show characteristic differences between the two species, such as the wavelength shift of the main blue maxima and the shoulder of *P*. *argus* at 320 nm. As a consequence, the two groups of points in S2 Fig, characterizing the positions of the specimens in the 3D visual space of the Lycaenid butterflies, are also well separated, despite the variability of each species. According to S2 Fig, *P*. *argus* exhibits more pronounced variation, irrespective of the operator.

The reason for the similarity in the naked eye examination (see Fig 1), despite the spectral dissimilarities, is that spectral differences at approximately 320 nm are not perceptible by human eyes (or with standard digital cameras) but an additional photoreceptor type in insect eyes is capable of efficiently detecting the spectral range between 300 and 400 nm [12], which results in different, species-specific wing coloration detection [11].

When investigating the color at the level of the individual, natural variability in the reflectance spectra can be observed, even between the wings of the same individual (Fig 4). To illustrate this more clearly, every spectrum measured on the wings of the two investigated species was plotted together in the background of the averaged curves (Fig 2), showing the varied wavelength of the main reflectance peaks of both species and the varied intensity ratio of the main peak and the shoulder in the UV in the case *P*. *argus* specimens. The differences in the spectra of Fig 2 towards the red side are attributed to the normalization of the reflectance spectra to the main peak in the blue region.

It is interesting to compare the averaged normalized spectra (Fig 2) and the averaged spectra without normalization (Fig 9A). For the non-normalized spectra, the UV side of both spectra exhibit a small shoulder on the left side of the main maximum. Since each of the curves is the result of averaging 100 individual measurements, accidental effects can be ruled out. Comparing the shape of the two normalized curves (Fig 2), the reflectance of *P*. *icarus* exhibits an almost symmetrical shape, whereas the left shoulder of *P*. *argus* indicates the presence of two overlapping maxima, and the right sides of the two curves are parallel. Fig 9A shows that in the range of the left side of the two overlapping maxima, the two curves are on top of each other and then start to deviate at the wavelength at which the contribution of the right side of the overlapping maxima becomes dominant. According to the optical model discussed in [35], this indicates that the nanoarchitectures of both *P*. *icarus* and *P*. *argus* contain two slightly different substructures with different perforation factors: one generating a reflectance maximum at the main blue peak and the other with a reflectance maximum in the range of the UV shoulder. The ratio of the two nanoarchitectures is different in the case of the two species: for *P*. *argus*, the two nanoarchitectures are present in almost similar quantities, whereas in *P*. *icarus*, the nanoarchitecture that reflects light at longer wavelengths is dominant. According to the numerical modelling reported in [35], the width of the reflectance band substantially narrows from approximately 200 nm for an unperforated multilayer (p = 0) to approximately 100 nm when the perforation factor is p = 0.4. This narrowing occurs in such a way that the shorter wavelength side of the reflectance maximum is fixed, while the longer wavelength side moves (see Fig 4 in [35]). Therefore, in the case of *P*. *argus*, different perforation occurs inside the scale layers.

### Accuracy of the data obtained by different optical setups

From an optical point of view, butterfly wings are complex objects. Even when the wing does not exhibit an intricate color pattern, the measured surface is covered by a mosaic of scales of approximately 100 x 50 μm^2^ in the case of Lycaenids (see Fig 10). Precise knowledge of the limitations and strengths of the light-collection method used on such a complex optical object is required to conduct careful interpretation of the spectral data.

The biological variability means that measurements performed on only a few specimens may be misleading. On the other hand, in most cases, the destruction of several tens of museum exemplars is unacceptable. Therefore, measurement methods that do not harm museum exemplars are needed [30]. Unfortunately, the methods that fulfil this condition may be more strongly influenced by the complexity of the object of study. To gain more insight into these effects, we investigated the detailed characteristics of different light-collection methods.

Variability of the dorsal coloration, in addition to the spectral position differences, appears in the amplitude variance of the peak in the blue wavelength region (Fig 5). The different amplitude variance of the perpendicular (Fig 5A) and integrating sphere (Fig 5B) spectra is the result of the difference in the method of light collection. The higher variance of the perpendicular detection is attributed to the complex surface properties of the butterfly wing, which generates significantly more angle-dependent reflection of the incident perpendicular light. These properties are defined on different length scales from a few hundreds of nanometers (scale level) to the micron scale (interscale level) and millimeter scale (wing scale). If high-intensity reflection of the illuminated scales directly reaches the perpendicular collecting optical fiber, then a spectrum with a high-amplitude blue peak can be recorded. The opposite case occurs when the illuminated scales scatter the light in the wrong, non-perpendicular direction, so only low amplitudes are detected. In contrast, the integrating sphere (Fig 5B) can eliminate this effect by collecting reflected light emerging under a wide angle range from the perpendicularly illuminated surface (in our case, from the whole upper hemisphere above the wing) and with a larger illumination spot, which results in lower variance of the spectral signals. The spectral positions of the blue reflectance maxima measured using the two different light-collecting techniques are coincident within 1 nm in the case of the averaged curves in Fig 5. If sufficiently large sample numbers are used, which is usually not the case in structural color investigations, then the two light-collecting methods yield similar spectral positions. However, when comparing measurements of single samples, differences of up to 40 nm and 30 nm, respectively, may be found for normal incidence measurements (Fig 6A) and integrated measurements (Fig 6B). This spread in the data does not originate from effects that are inherent to the measurement principle or the instrument but as a consequence of differences in the measured objects.

The accuracy differences of the two measurements were shown using histograms of the amplitude and peak position (Fig 6): the measured amplitude variance of the reflectance spectra increased significantly when the perpendicular light-collecting method was applied. The spectral position variance required further analysis using the chromaticity diagram of the polyommatine butterflies, resulting in differently distributed chromaticity points in 3D color space (see the details in S1 File).

The results related to the accuracy of the two optical measurement setups show that for the investigation of the intra-species variability of structural coloration (arising from eventual geographic, climatic, or applied experimental conditions), the integrating sphere technique is more suitable. Despite the higher variance of the collected data with the perpendicular measurement setup, it has important advantages over the integrating sphere light-collecting method, which was exploited in our previous experiments: the perpendicular probe can be used for rapid, non-destructive reflectance measurements without harming fragile exemplars, which is important when set museum specimens are investigated [30]. Another important factor is that when measuring a single specimen, both the spectral position and the magnitude of the reflectance peak may be affected by the biological variability of the specimen.

### Reproducibility of measurements conducted by different human operators

The reproducibility of the optical measurements is key when minor color differences are investigated, as in the case of the color variability of polyommatine Blues. The integrating sphere light-detection showed significantly lower variance in the measured data compared to the perpendicular measurements, which is related to the optically complex surface of the butterfly wings (Fig 10). In Fig 7, both *Polyommatus icarus* and *Plebejus argus* were investigated in terms of the amplitude and spectral position variance in the independent measurements of two operators on different days. Between the two measurement sessions, the experimental setups were taken apart and reassembled to simulate measurements performed in different laboratories. For both investigated species, the histograms of the spectral parameters measured by the two operators were reproducible. Fig 7 shows only minor differences between the amplitude and spectral position histograms of *P*. *icarus* and *P*. *argus* specimens measured using the integrating sphere light-collecting technique. The wavelength ranges over which the spectral positions were extended are coincident; some deviations were found in the number of individual samples within a certain bin. The measured spectra were transformed and compared in the 3D chromaticity diagram (see S2 Fig). Both results demonstrate the high degree of similarity of the data sets when the two independent measurements were conducted. Furthermore, when using the integrating sphere, the detailed investigation of the peak width (FWHM) in the blue wavelength region (Fig 8) shows the high degree of reproducibility of the sample related variation of the structural coloration over 100 samples and strongly supports that the differences measured on individual specimens are characteristic of those specimens and are not a result of measurement error.

The results confirm that the integrating sphere spectral measurement setup has higher reproducibility and is less affected by the operator; therefore, it is suitable for the rigorous investigation of small optical differences, like the structural color variability in Blue butterflies (see S1 File). Additionally, these data demonstrate that the colors of the two butterfly species are well separated in 3D color space and have distinct colors when observed by butterflies. On the other hand, when the available samples, for example, museum specimens, do not allow the removal of the wings, normal incidence measurements, if averaged over a sufficiently large sample batch, may be used.

### Color intensity differences and variability of the two species

The integrating sphere reflectance measurements showed spectral differences between the species (Fig 2); a slight shift of the main maximum in the blue region and a shoulder in the near UV wavelength range were observed. Accordingly, the separated data points in the 3D chromaticity diagram (see S2 Fig) showed a distinction in the two hues of blue on the wings of these species. The intensity characteristics of the two species are presented separately in Fig 9A by averaging the measured reflectance curves.

Brighter wing coloration of *Polyommatus icarus* males is observed in Figs 1 and 9A compared to *Plebejus argus* specimens. This may be a direct consequence of the different prezygotic mating strategies of the two species. The patrolling males of *P*. *icarus* execute approximately 20 wingbeats per second while in flight [36]. Taking into account Fig 3, this means that relative to a given observer, their wings are in a suitable position to generate reflected color for only a few fractions of a second. Additionally, due to the patrolling, they may be at a distance of several meters to tens of meters away. They have to be more conspicuous for sufficient visibility by females. The perching mating strategy of *P*. *argus* males, which involves lekking in their microhabitats, requires a less intense blue color because the females identify the males in a fixed position and from a significantly shorter distance.

In contrast to the intensity of the coloration, the structural color variability of the two species showed similar characteristics: the Gaussian-like behavior of the amplitude variance histograms (Fig 9B and 9C, left panels) and the sharp cut-off at ±10 nm of the spectral position histograms (Fig 9B and 9C, right panels) were almost identical. The high degree of similarity between the amplitude and spectral position distributions of the two species indicate that the structural color variability is independent of the prezygotic mating strategy. The deviation from the Gaussian shape, i.e., the sharp edges of the spectral position distributions, which indicate that no individuals with outlier coloration were observed, show that the color composition of the local butterfly fauna is limited by color-based conspecific recognition. The genes of individuals with the “wrong” color likely have a lesser chance to be transmitted to the following generation; therefore, limited color variability is viable.

Finally, when comparing the spread of amplitudes within the species *P*. *icarus* in Fig 6A with those in Fig 6B, i.e., the spread of normal incidence measurements with the spread of those taken with the integrating sphere, some differences are noted: while the spread of the integrated data (angle-independent measurement) is symmetrical, the spread of the normal incidence data has a long tail towards the side of high-reflectance individuals. This may indicate that the more brilliant males—with an angularly more ordered arrangement of their scales—despite their comparatively lower numbers, are more successful in transmitting their genes than the less brilliant males.

## Conclusions

In summary, the rigorous examination of the coloration of all four wings of 25 male butterflies (4 x 25 = 100 samples) from the species *Polyommatus icarus* and *Plebejus argus*, both possessing iridescent structural color on their dorsal wings, convincingly shows that within the species, the maximum of the reflectance spectra may deviate by ±15 nm when measured by integrating sphere and by as much as ±20 nm when measured by a normal incidence setup. Moreover, even when measured with the integrating sphere, the four wings of the same individual may exhibit differences. The two forewings and the two hindwings are usually more similar, and the forewing-hindwing differences are usually larger.

The larger deviation of the normal incidence setup is attributed to the deviation of the orientation of the individual scales with respect to the wing membrane and to minor structural differences between individual scales, each of which is secreted by different, specialized cells. Despite this, the colors of the two species form two well-separated clusters in 3D color space (see S1 File), which is a consequence of the shoulder on the UV side of the reflectance maximum in the case of *P*. *argus*. The significantly more pronounced UV shoulder for *P*. *argus* is attributed to a higher fraction of layers with a larger perforation factor than in the scales of *P*. *icarus* specimens.

The comparison of the measurement sets of two independent operators shows that the measurement itself is highly reproducible (Figs 7 and 8 and S2 Fig); the measured deviations may be attributed to the natural biological variability of the nanoarchitectures and of the color they generate. If high-accuracy comparison of the coloration of different populations or of groups of individuals that have been subjected to special conditions that may induce modifications of color is to be achieved, a sufficiently large number of individuals is required, and if possible, the use of reflectance measurements with an integrating sphere is preferable.

The comparison of data averaged over 100 samples indicates that the prezygotic mating strategy, patrolling for *P*. *icarus* and perching for *P*. *argus*, may influence the intensity of the sexual signaling color used by the two species. The deviation in the color distribution from a Gaussian distribution for both species, like that found in the deviation of the spectral positions of the reflectance maxima, shows that the use of color in sexual recognition limits the allowed color deviations.

## Supporting Information

S1 Fig (A) Chromaticity diagram results and (B) the radial distribution functions of the two optical measurement setups. (A) The integrating sphere chromaticity points (n = 200) are localized in a smaller area, and the perpendicular measurement results (n = 200) have higher variance. (B) The histogram of the integrating sphere chromaticity points shows smaller deviation of the measured distance than the perpendicular measurement setup.
(TIF)

S2 Fig 3D chromaticity diagram results of two independent integrating sphere measurements of *Polyommatus icarus* and *Plebejus argus* males. The high reproducibility of the measurement method can be observed as the clusters of the two species’ chromaticity points assemble into similar positions and shapes in the two measurements (n = 200 each). The color differences of the two species can also be observed: the shoulder at 320 nm of *P*. *argus* specimens (see Fig 2) produces good separation in the spectral data in the butterfly color space, which also means that the structural blue coloration of these butterflies is species-specific; thus, it is suitable for color-based sexual communication.
(TIF)

S1 File Spectral analysis using the chromaticity diagram of the polyommatine butterflies. (DOCX)
