# Supplementary figures and images for: Variability of the Structural Coloration in Two Butterfly Species with Different Prezygotic Mating Strategies
Source: PLoS One. 2016 Nov 10;11(11):e0165857. doi: 10.1371/journal.pone.0165857 (PMC5104395; doi:10.1371/journal.pone.0165857)

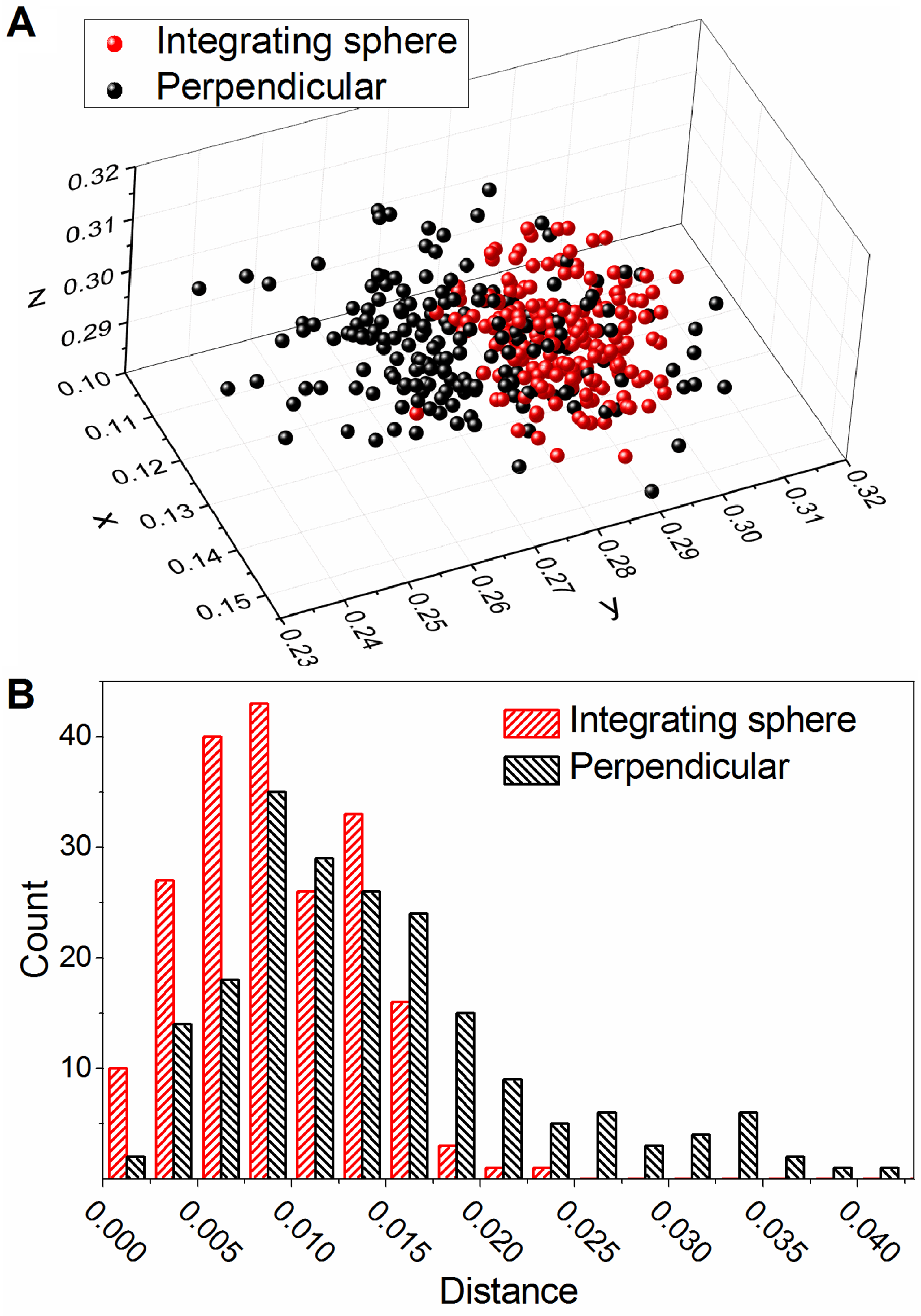

Supplement: S1 Fig — (A) The integrating sphere chromaticity points (n = 200) are localized in a smaller area, and the perpendicular measurement results (n = 200) have higher variance. (B) The histogram of the integrating sphere chromaticity points shows smaller deviation of the measured distance than the perpendicular measurement setup. (TIF) [file pone.0165857.s001.tif]

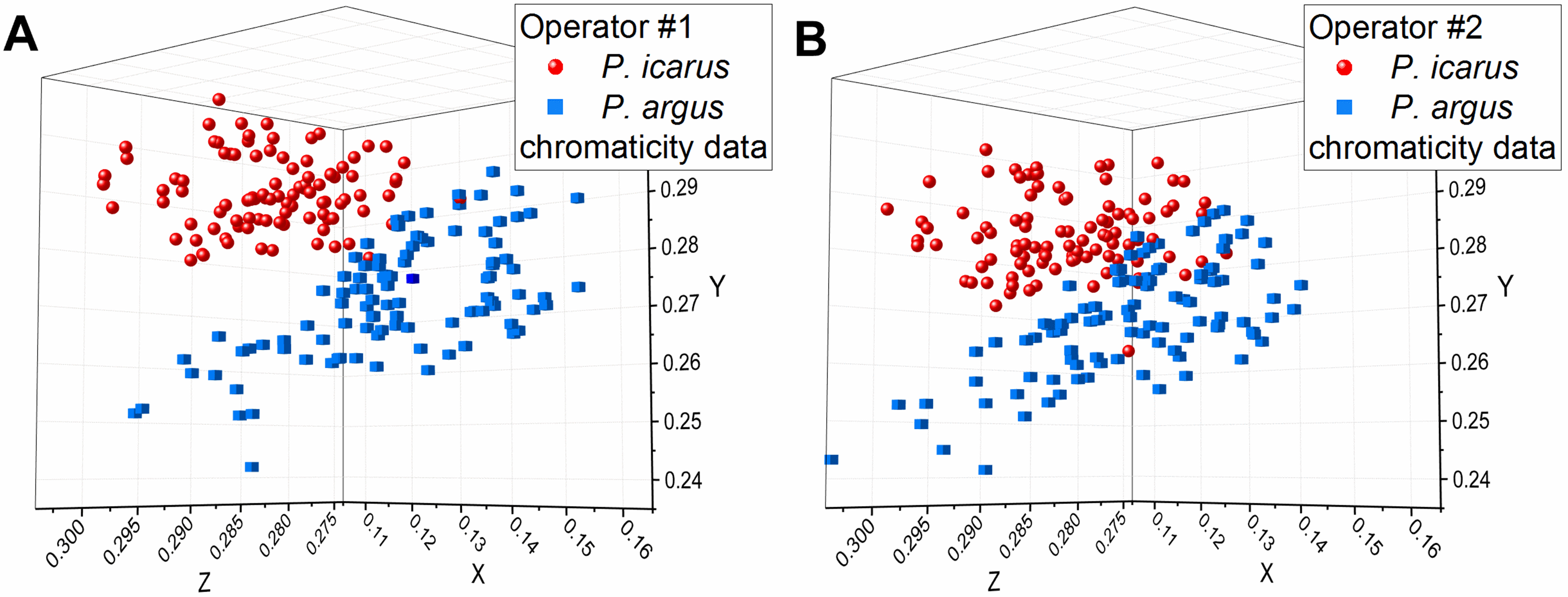

Supplement: S2 Fig — The high reproducibility of the measurement method can be observed as the clusters of the two species’ chromaticity points assemble into similar positions and shapes in the two measurements (n = 200 each). The color differences of the two species can also be observed: the shoulder at 320 nm of P. argus specimens (see Fig 2) produces good separation in the spectral data in the butterfly color space, which also means that the structural blue coloration of these butterflies is species-specific; thus, it is suitable for color-based sexual communication. (TIF) [file pone.0165857.s002.tif]
