# Supplementary material for: Variability of the Structural Coloration in Two Butterfly Species with Different Prezygotic Mating Strategies
Source: PLoS One. 2016 Nov 10;11(11):e0165857. doi: 10.1371/journal.pone.0165857 (PMC5104395; doi:10.1371/journal.pone.0165857)
Supplement: S1 File — (DOCX) [file pone.0165857.s003.docx]

**Supporting Information**

**Spectral analysis**

In the case of the reflectance spectra, a simple description of the peak shape is given by its width by measuring the full width at the half maximum (FWHM) (Fig 8). To measure accurate FWHM values, the tangential baseline was subtracted from each spectrum, as described in [10]. For the comparison of the UV-VIS-NIR spectra, we developed a method similar to the CIE 2D chromaticity diagram to represent each spectrum as a single point in 3D color space by taking into account the four types of visual pigments in butterfly eyes [11] (S1 and S2 Figs).

To analyze the effect of spectral position deviations occurring when different measurement setups were used (see the right panels in Figs 4A and 4B), the chromaticity diagram of the polyommatine butterflies was applied [11], and a 3D diagram containing the chromaticity data points was obtained (S1A Fig) to facilitate the comparison. Each of the 100 reflectance spectra generated a single point in this chromaticity diagram, which represents a hue in the color space of the polyommatine butterflies. The chromaticity points of the different measurement setups cover different areas of the diagram: the points of the integrating sphere measurement (red points) are concentrated in a smaller area than the perpendicular measurement data (black dots), and the latter also have higher variance. To demonstrate this quantitatively, circles were fit to both measurement data sets (centered on the geometric center of the distribution of points), and the radial distribution functions of the data points measured from the centers of the circles were generated, as shown in S1B Fig. The histograms indicate higher variance of the perpendicular data, and the character of the distribution alters slightly between the two light-collection methods: the more distant data points have minor, non-zero occurrence, while the less distant distribution of normal incidence data remains similar to the integrating sphere result.


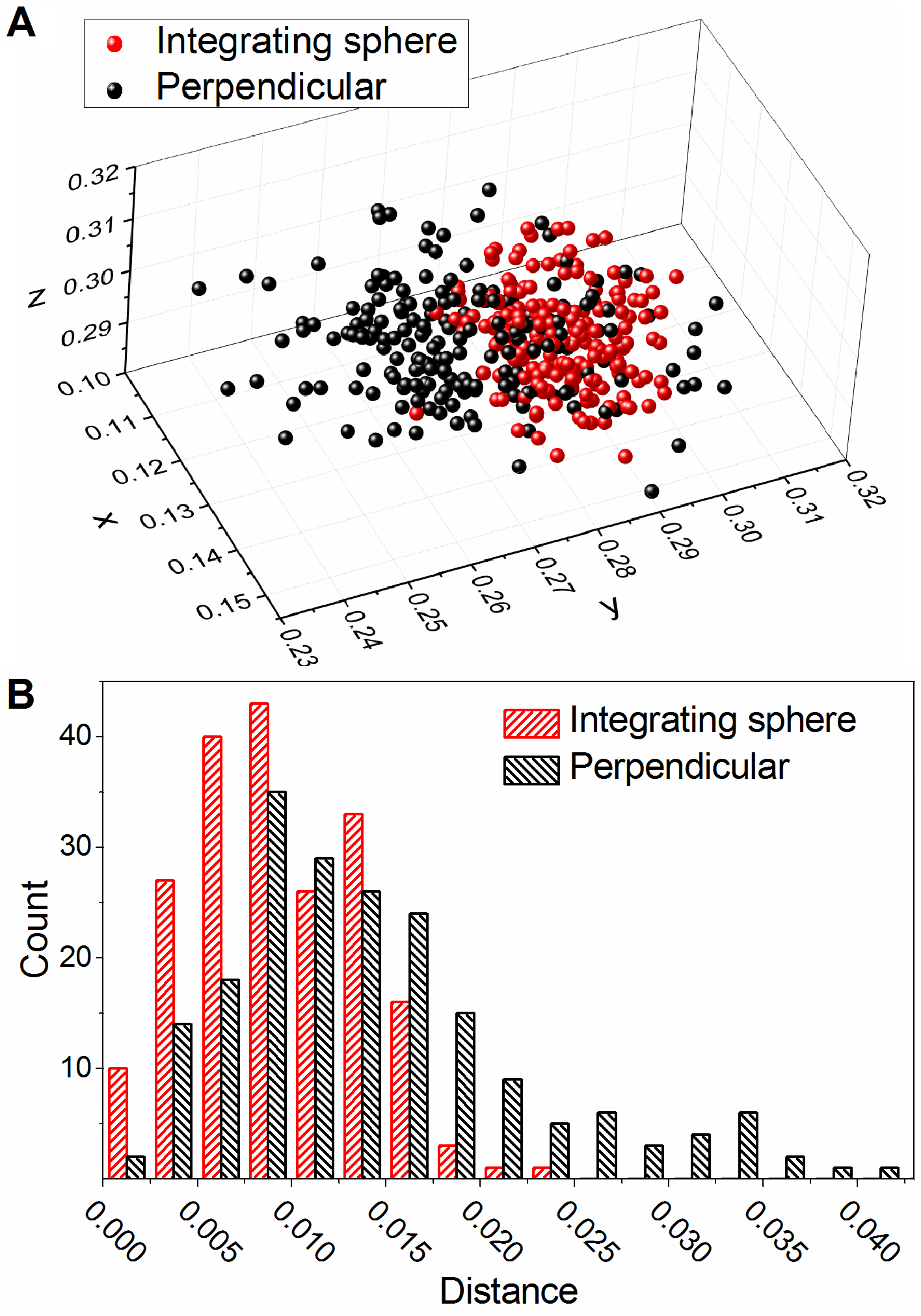


**S1 Fig. (A) Chromaticity diagram results and (B) the radial distribution functions of the two optical measurement setups.** (A) The integrating sphere chromaticity points (n=200) are localized in a smaller area, and the perpendicular measurement results (n=200) have higher variance. (B) The histogram of the integrating sphere chromaticity points shows smaller deviation of the measured distance than the perpendicular measurement setup.

The areas covered by the two sets of points in S1A Fig have different patterns of scattering. While the data measured with the integrating sphere have a circular spread, the data measured with the normal incidence setup have an elliptical shape. The minor axis of the ellipse is of the same magnitude as the diameter of the circular spreading pattern, which suggests that the additional scattering in one direction originates from light falling under slightly different angles of the scales on different wings. The scales do not lie exactly in the wing plane; they make a certain angle of the order of 20° with the wing membrane (Fig 10A). This factor, together with a few degrees of rotation around the cover scale axis (Fig 10B), may be responsible for the larger deviation in the perpendicular data (the scale is not rigidly fixed in the scale membrane but through a narrow pedicel).

The reproducibility of the spectral results can be analyzed using the chromaticity diagram of the polyommatine butterflies. We show the chromaticity points of the two species measured independently by two operators. The reflectance spectra were transformed and plotted in 3D color space (S2 Fig), with each point corresponding to a full spectrum measured on one of the 100 samples. The data points aggregate into similar clusters for both species, and the shape and the distribution of the clusters remain almost unchanged, irrespective of which operator performed the measurements.


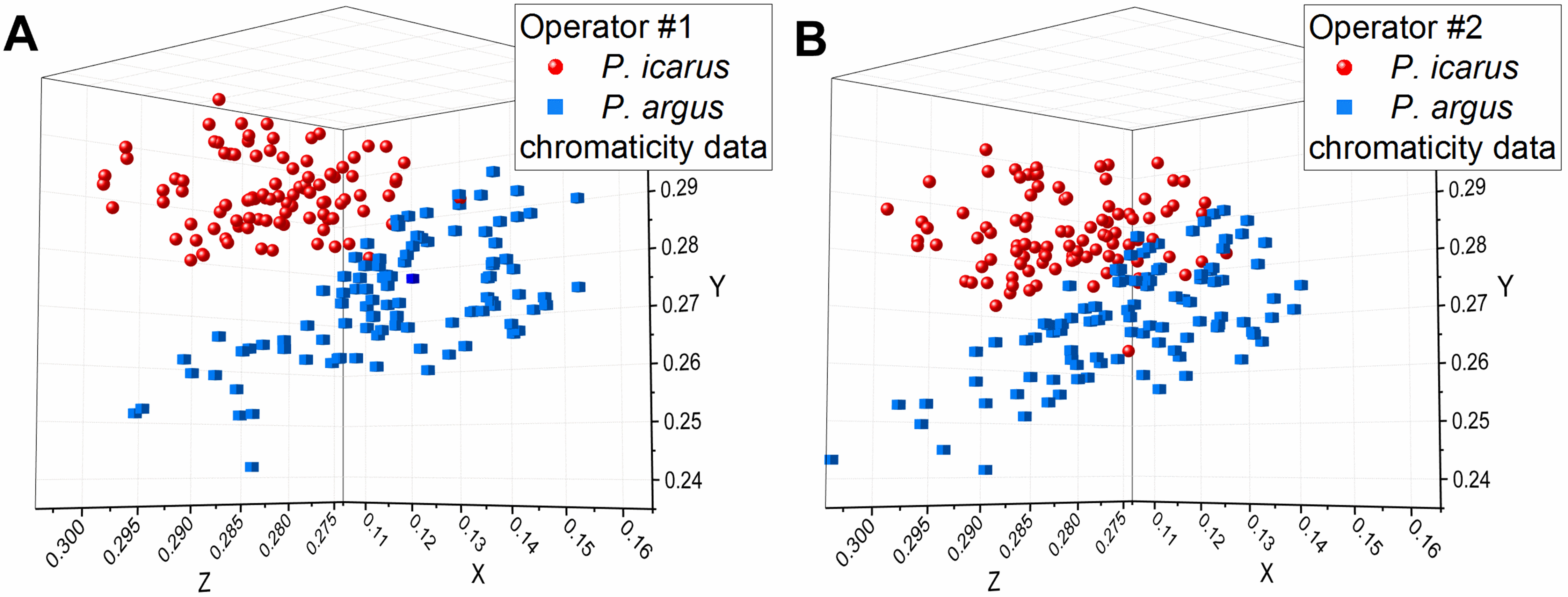


**S2 Fig. 3D chromaticity diagram results of two independent integrating sphere measurements of *Polyommatus icarus* and *Plebejus argus* males.** The high reproducibility of the measurement method can be observed as the clusters of the two species’ chromaticity points assemble into similar positions and shapes in the two measurements (n=200 each). The color differences of the two species can also be observed: the shoulder at 320 nm of *P. argus* specimens (see Fig 2) produces good separation in the spectral data in the butterfly color space, which also means that the structural blue coloration of these butterflies is species-specific; thus, it is suitable for color-based sexual communication.
